# Surgical management of infected endovascular aortic stent graft secondary to *Coxiella burnetii* infection

**DOI:** 10.1016/j.jvscit.2024.101581

**Published:** 2024-07-23

**Authors:** Sai Konda, Daniel Ihnat, Paul Orecchia

**Affiliations:** a Division of Vascular Surgery, Department of Surgery, University of Minnesota Medical Center, Minneapolis, MN; b Division of Vascular Surgery, Minneapolis VA Medical Center, Minneapolis, MN

**Keywords:** Aortic graft infection, Cryopreserved allograft, Chronic Q fever, *Coxiella burnetti*

## Abstract

The management of an endograft infection with *Coxiella burnetti* (Q fever) is presented. In this case report, we describe a unique case of an endovascular aneurysm repair (EVAR) that was originally placed for a 6.4-cm abdominal aortic aneurysm with an aorto-left renal vein fistula. In retrospect, the abdominal aortic aneurysm was most likely infected at the time of EVAR. The patient presented 2 years later with a virulent infection of the stent graft requiring explantation and reconstruction. This case highlights surgical management of an infected EVAR with homograft reconstruction and subsequent antibiotic management.

Aortic graft infections (AGIs) are uncommon, ranging from 0.2% to 2.7%.^1^^,^^2^ AGIs are usually caused by gram-positive, gram-negative bacteria and occasionally fungal or mycobacterial species. An uncommon cause is *Coxiella burnetti* (Q fever), which may be present before abdominal aortic aneurysm (AAA) repair, especially when a fistula is present. This case report highlights the indolent presentation of chronic Q fever and illustrates the difficulties in the diagnosis, the surgical and antibiotic management. The patient consented to publication of this report.

## Case report

**Fig 1:**
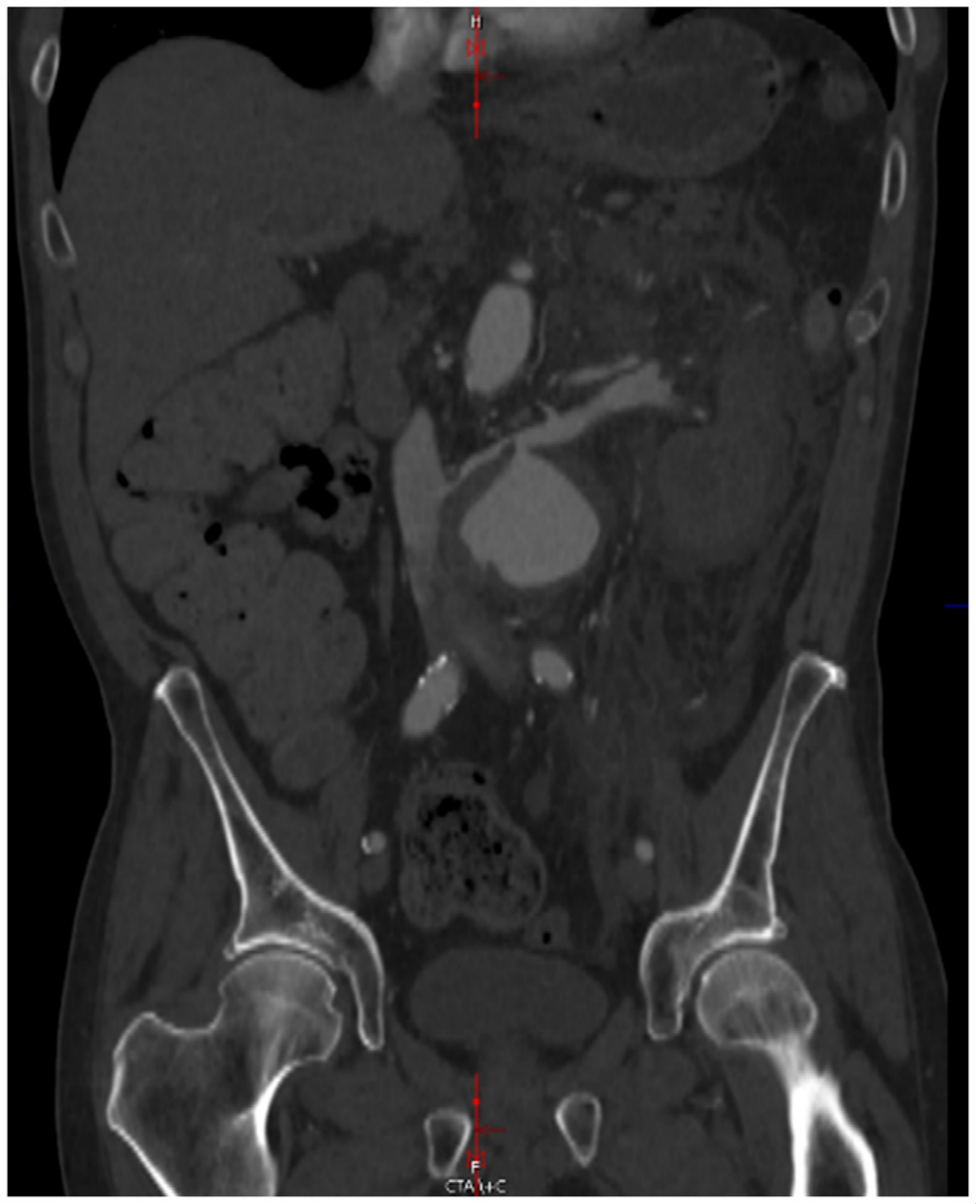
Original imaging study demonstrating aorto-left renal vein fistula.

**Fig 2:**
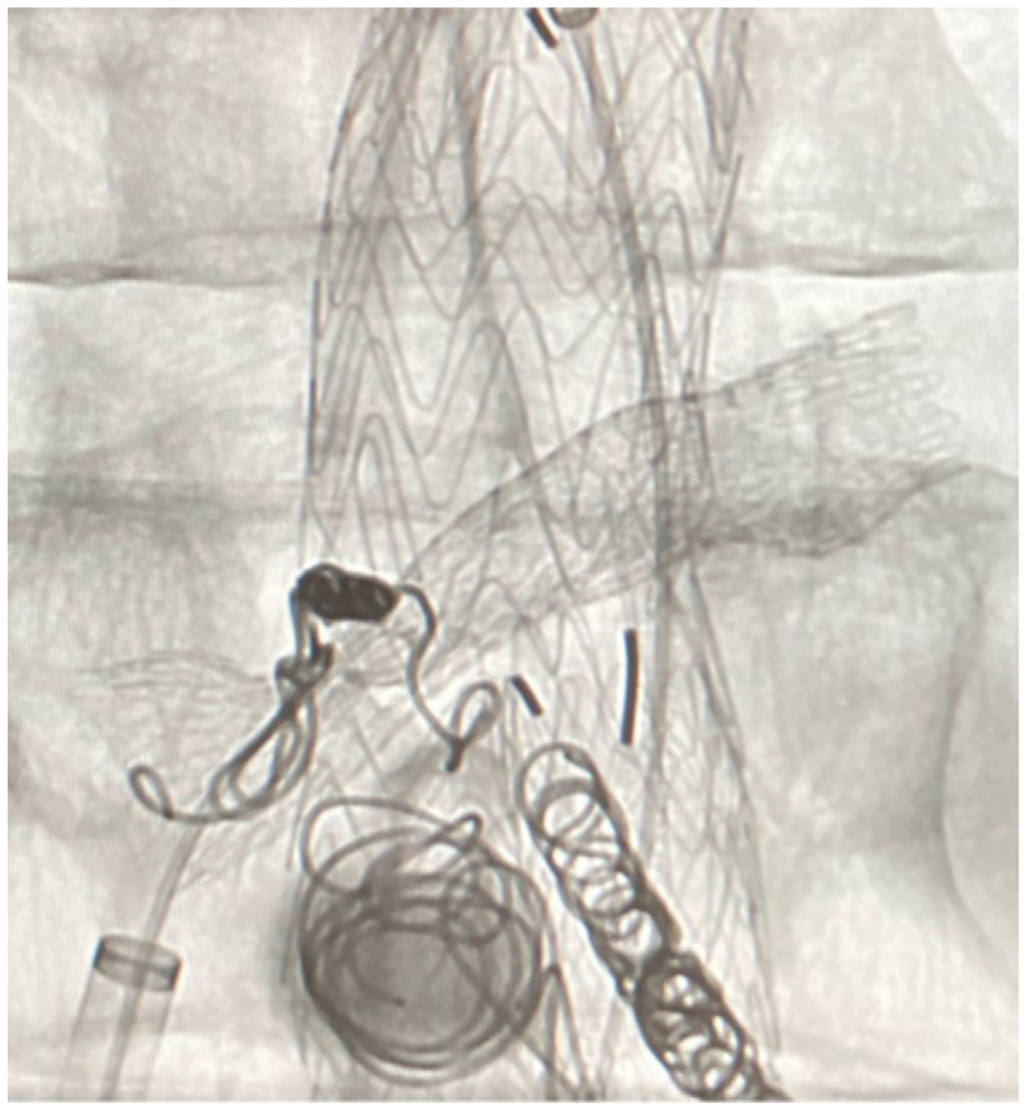
Follow-up procedure at which time lumbar and inferior mesenteric arteries are coiled and a covered stent is placed in the left renal vein for management of type II endoleak.

**Fig 3:**
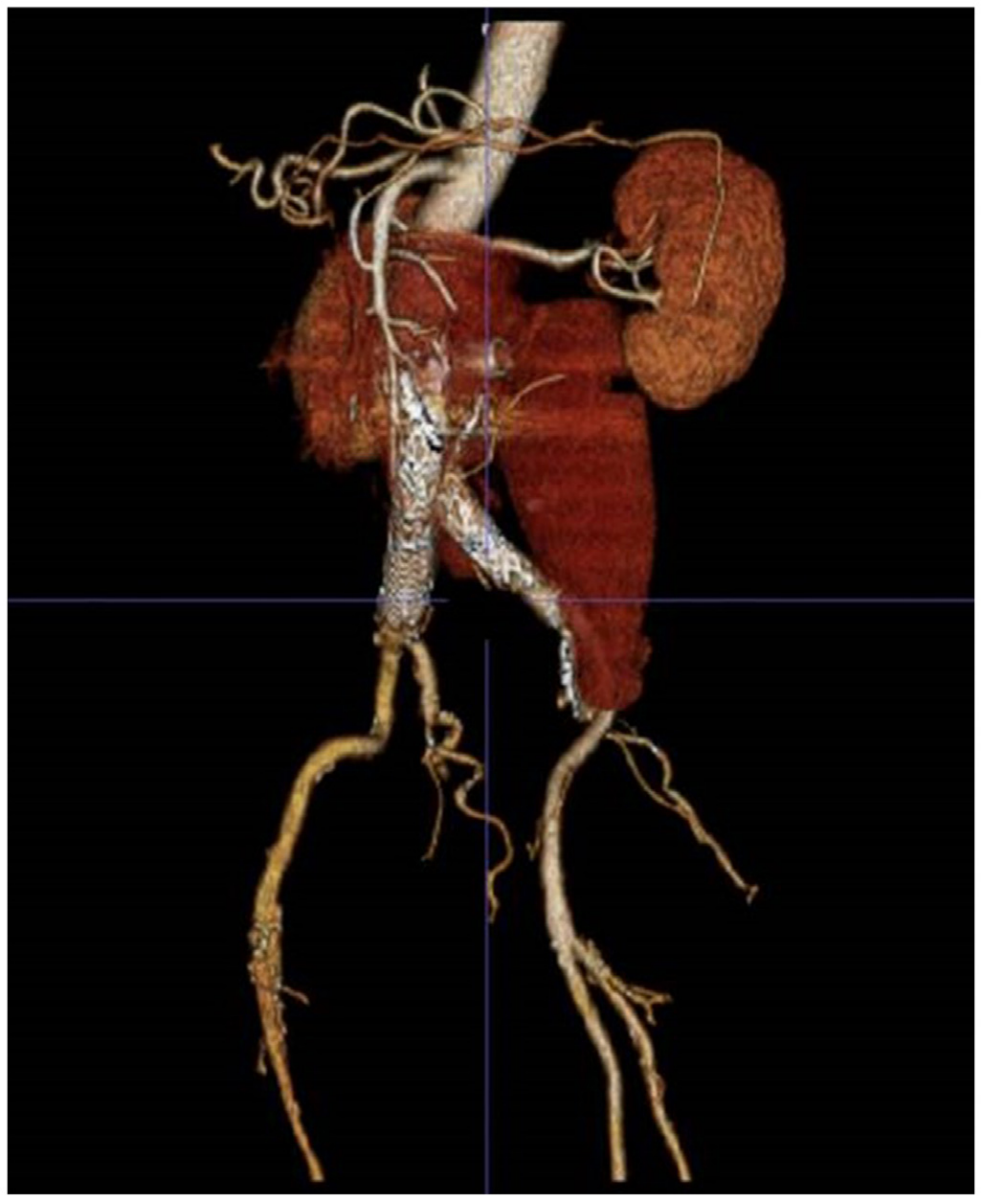
Three-dimensional reconstruction of a computed tomography scan demonstrating air and fluid adjacent to the aortic stent graft.

A 63-year-old gentleman had initially presented with abdominal and back pain, and was found to have a 6.4-cm AAA with an aorta to retro-aortic left renal vein fistula (AVF) (Fig 1). The patient lives in rural Wisconsin, and has daily contact with his brother-in-law, a dairy cattle hoof trimmer. The patient underwent endovascular aneurysm repair (EVAR) using a Gore (W. L. Gore & Associates, Flagstaff, AZ) bifurcated endograft. One month later, he underwent coil embolization of the lumbar and inferior mesenteric arteries and placement of a covered stent in the left renal vein for a persistent type II endoleak and aorto-left renal vein AVF (Fig 2). Two years later, the patient presented to our hospital with a 6-month history of malaise, anorexia, 40-pound weight loss, and worsening left sided back pain. Computed tomography scan revealed rim-enhancing fluid collections adjacent to the endograft (Fig 3). He was started on empiric vancomycin and piperacillin-tazobactam. Percutaneous drainage produced purulent fluid. He remained afebrile, with a normal white blood cell count, and negative blood and fluid cultures. The clinical picture was consistent with AGI and we prepared him for graft explantation.

**Fig 4:**
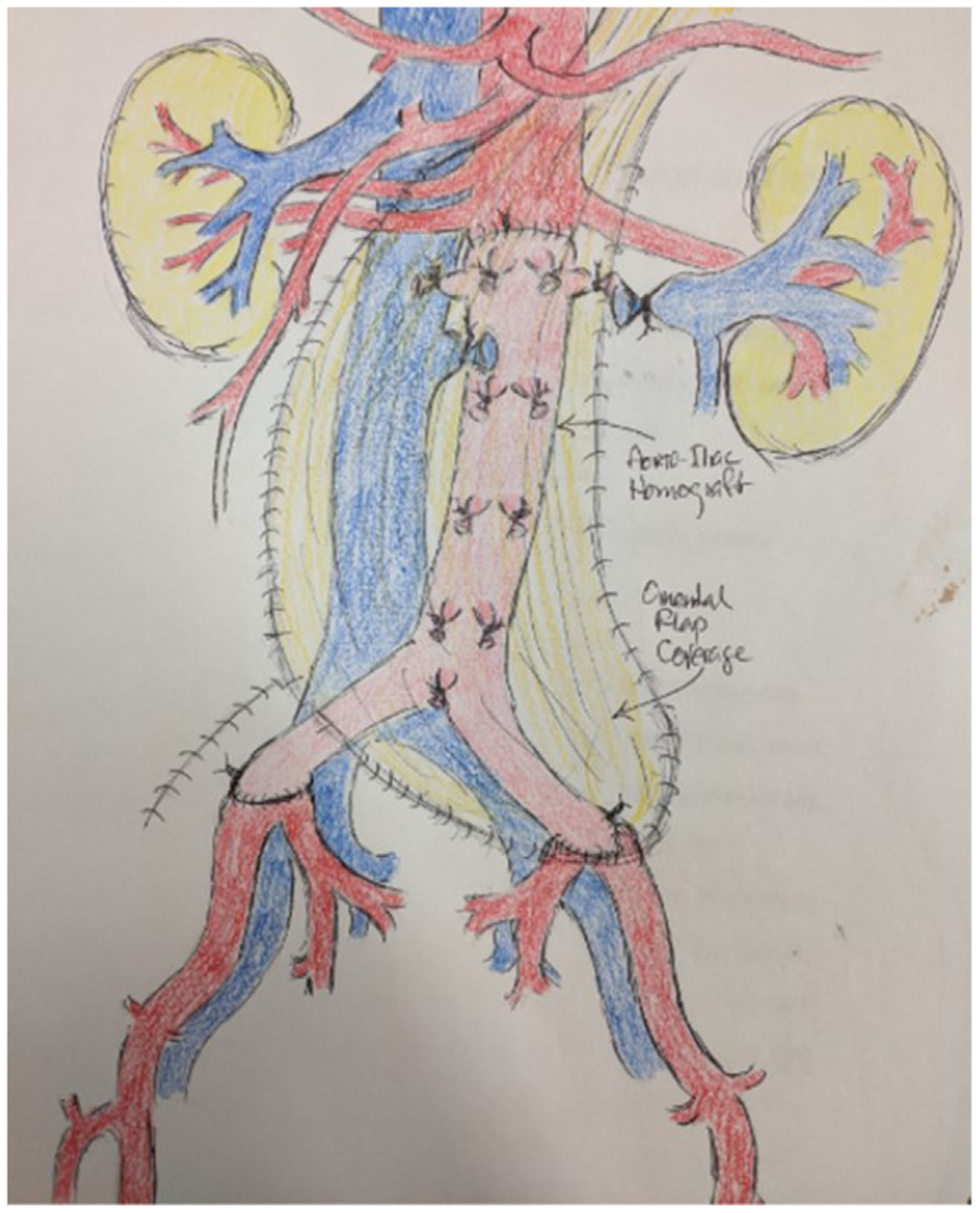
Postoperative schematic. Homograft repair with explantation of previous aortic stent graft and Viabahn stent and pedicled omental patch.

The aorta was exposed through a midline incision. An inflammatory mass extended along the left psoas muscle from the pelvis to the left renal artery. We exposed the paravisceral aorta with loop control of the superior mesenteric artery and both renal arteries. A proximal cross-clamp was placed in between the celiac and the superior mesenteric artery and the distal cross-clamp was placed at the common iliac bifurcations bilaterally. Aortotomy was performed with evacuation of a large amount of purulent fluid. The prior aortic endograft, renal vein covered stent, and all coils in the sac were explanted. The renal vein was oversewn medially and laterally with preservation of collaterals. There was insufficient room to safely oversew an infrarenal aortic stump without compromising renal artery perfusion. We felt that aortic homograft replacement was the best reconstruction option to expedite and simplify the procedure in a nutritionally compromised patient. In-line replacement with a homograft is our preferred approach for AGI. The homograft was anastomosed to the juxtarenal aorta (cross-clamp time, 21 minutes) and distally to the bilateral common iliac artery bifurcations. After additional debridement, the retroperitoneal area was free of gross infection, and an omental pedicled flap was sutured over the homograft (Fig 4). Several deep lumbar and inferior mesenteric artery coils were unable to be removed safely.

**Fig 5:**
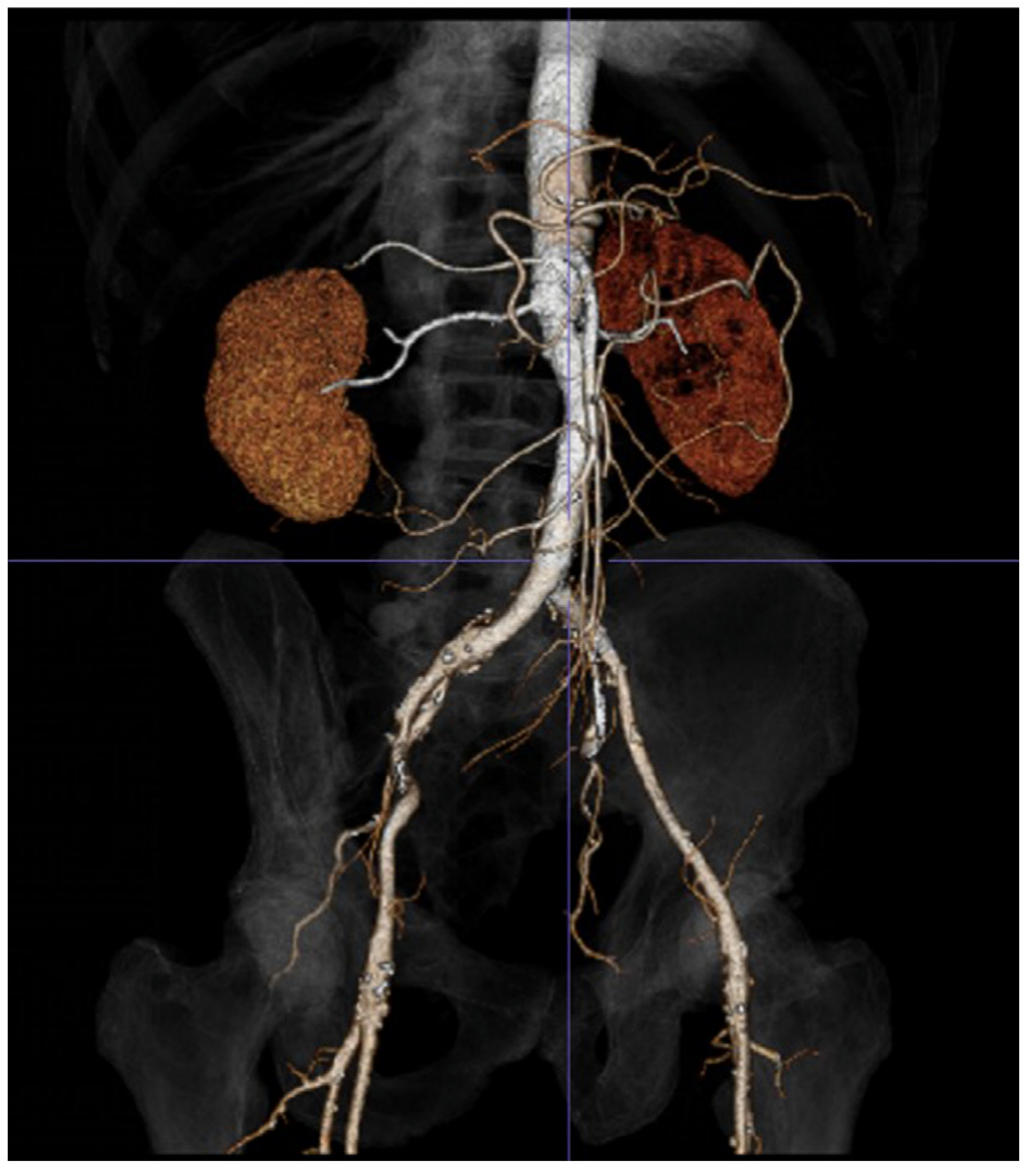
Postoperative computed tomography scan demonstrating patent stent graft at 33 months.

The patient had a routine postoperative course and was discharged home on postoperative day 7. All tissue and blood cultures were negative after 7 days, and gram stains of the aortic wall and perigraft fluid demonstrated white blood cells only. One specimen of the aortic wall was sent for polymerase chain reaction testing of the 16S rDNA sequence and demonstrated *C burnetti*, which was reported 2 days after discharge. His Q fever titers were markedly elevated (Table I). The patient completed an 18-month course of doxycycline 100 mg twice daily and hydroxychloroquine 300 mg twice daily and then discontinued antibiotics. At the 1 year follow-up, the patient had return of appetite, appropriate weight gain, and no evidence of infection. At 33 months, his Q fever IgG titers are markedly decreased, consistent with his previous history of chronic Q fever infection (Table II). He underwent Computed tomography at 33 months which demonstrated an unremarkable reconstruction (Fig 5). He continues to do well 58 months after endograft removal.

**Table I:** Initial postoperative Q fever titers

Test	Result	Reference range
Q fever IGM	Positive	
Q fever IgG phase 1	Positive	
Q fever IgG P1 titer	1:16,384	Ref: <1:16
Q fever IgG phase 2	Positive	
Q fever IgG P2 titer	1:16,384	Ref: <1:16

**Table II:** 33-Month postoperative Q fever titers

Test	Result	Reference range
Q fever IgM	Negative	
Q fever IgG phase 1	Positive	
Q fever IgG P1 titer	1:1024	Ref: <1:16
Q fever IgG phase 2	Positive	
Q fever IgG P2 titer	1:2048	Ref: <1:16

## Discussion

*C burnetti* AGI are rare but described.^3^^,^^4^ The treatment requires surgical explantation combined with more protracted treatment of chronic Q fever. *C burnetti* is a gram-negative intracellular bacterium that causes Q fever. It is usually transmitted from livestock via aerosols from contaminated waste, ingestion, or tick bites. Q fever is categorized into acute and chronic forms. Acute Q fever usually manifests as a self-limited flu-like illness and can be associated with pneumonia or hepatitis. Chronic Q fever can present as endocarditis, aortic aneurysm, AVF, or AGI. Approximately 1.5% to 11.0% of acute Q fever cases will evolve to chronic Q fever.^5^^,^^6^

The largest Q fever outbreak ever documented estimated 40,000 infections from 2007 to 2010.^3^^,^^4^ Vascular infections occurred in 169 patients, 26 (15%) of whom developed AVF. In our patient, we believe his aorta was infected at the time of EVAR, because his aorta-left renal vein AVF was present. The overall prognosis for patients who present with Q fever related AVF is poor with mortality ranging from 24% to 60%.^3^^,^^4^ Patients with a vascular prosthesis or an arterial aneurysm have a 25-fold increased risk for progressing to chronic Q fever.^4^

The surgical management of AGIs requires removal of the prosthetic graft with debridement of infected tissue and restoration of the circulation. This can be accomplished in several ways. Traditionally, it involved graft excision, oversewing of the aortic stump and axillobifemoral bypass. The downside of this method is an associated 3% to 22% risk of subsequent aortic stump rupture and potentially less long-term patency of the axillobifemoral bypass.

In situ reconstructions can be done using either rifampin-soaked Dacron grafts, aortic homografts, or the neo-aortic-iliac system procedure with harvested femoral veins. The neo-aortic-iliac system has excellent results with a procedure-related mortality of 14% and a cumulative primary patency of 82% at 72 months.^7^ Cryopreserved homograft has the advantage of not requiring harvesting. In the largest US study (222 patients) of cryopreserved homografts, the freedom from graft-related complications, graft explant, and limb loss was 80%, 88%, and 97%, respectively, at 5 years with a primary graft patency of 97%.^8^ Finally, in situ reconstruction with a rifampin-soaked Dacron graft and omental wrap is an option, but is best in patients with known low virulence infections (eg, *Staphylococcus epidermidis*).^9^^,^^10^ In this case, a cryopreserved homograft was used because it allowed for an expeditious in situ reconstruction with an acceptable durability.

Currently, there are no clear guidelines for antibiotic treatment for vascular Q fever. Management of vascular Q fever has been extrapolated from the treatment of Q fever endocarditis. An acceptable consensus for duration of antibiotics is at least 18 months at which time *C burnetii* antibody titers should have decreased by 4-fold.^11^ Eldin et al^12^ proposed a prophylactic strategy to decrease the incidence of AGI or primary AAA infections, which consists of 12 months of treatment with hydroxychloroquine and doxycycline. Furthermore, patients over the age of 65 diagnosed with Q fever should undergo a computed tomography scan or ultrasound examination to screen for AAA.^12^ They also report the use of positron emission tomography with computed tomography to detect early signs of infection.^12^

In conclusion, our case involved initially a primary aorto-left renal vein AFV, originating from an aortic aneurysm infected by *C burnetti*. This was not recognized as an infectious problem and the patient underwent EVAR and then left renal vein stent placement, which subsequently lead to an AGI requiring explantation, reconstruction, and an 18-month course of doxycycline and hydroxychloroquine. He is now 58 months postoperative; he has no evidence of ongoing infection and a functioning vascular reconstruction.

## Disclosures

None.
